# Mineralocorticoid receptor-antagonism prevents COVID-19-dependent glycocalyx damage

**DOI:** 10.1007/s00424-022-02726-3

**Published:** 2022-07-22

**Authors:** Benedikt Fels, Sovon Acharya, Carl Vahldieck, Tobias Graf, Nadja Käding, Jan Rupp, Kristina Kusche-Vihrog

**Affiliations:** 1 Institute of Physiology, University of Lübeck, 23562 Lübeck, Germany; 2 DZHK (German Research Centre for Cardiovascular Research), Partner Site, Lübeck, Germany; 3 Department of Anaesthesiology and Intensive Care Medicine, University Hospital Schleswig-Holstein/ Campus Lübeck, 23538 Lübeck, Germany; 4 University Heart Center, University Hospital Schleswig-Holstein/Campus Lübeck, 23538 Lübeck, Germany; 5 Department of Infectious Diseases and Microbiology, University of Lübeck, 23538 Lübeck, Germany

## Abstract

Proinflammatory cytokines target vascular endothelial cells during COVID-19 infections. In particular, the endothelial glycocalyx (eGC), a proteoglycan-rich layer on top of endothelial cells, was identified as a vulnerable, vasoprotective structure during infections. Thus, eGC damage can be seen as a hallmark in the development of endothelial dysfunction and inflammatory processes. Using sera derived from patients suffering from COVID-19, we could demonstrate that the eGC became progressively worse in relation to disease severity (mild vs severe course) and in correlation to IL-6 levels. This could be prevented by administering low doses of spironolactone, a well-known and highly specific aldosterone receptor antagonist. Our results confirm that SARS-CoV-2 infections cause eGC damage and endothelial dysfunction and we outline the underlying mechanisms and suggest potential therapeutic options.

## Introduction

In addition to manifesting as an acute respiratory infection that may progress rapidly to acute respiratory distress syndrome (ARDS), it was recognized early on that SARS-CoV-2 infections exhibit a strong systemic inflammatory process that may lead to endothelial dysfunction and cardiovascular disease [1, 2]. Clinically, COVID-19-dependent endothelial dysfunction might contribute to pulmonary edema, proteinuria, and vascular inflammation and seems to be a central cause of multiorgan failure [3, 4]. Under healthy conditions, the vascular endothelium provides a crucial interface between blood and tissue. Due to this strategic position, it acts as a vasoprotective barrier in many different pathological conditions [5]. In particular, the endothelial top surface layer, the endothelial glycocalyx (eGC)—a negatively charged brush-like 0.5- to 1-µm-thick layer of membrane-bound, carbohydrate-rich molecules mostly comprising glycoproteins and proteoglycans—can be seen as a key player in vasoprotective function [6, 7]. Recently, Rovas and colleagues demonstrated that the thickness of the sublingual eGC, a robust and reliable marker for eGC integrity, was drastically decreased in COVID-19 patients compared to controls [8, 9], indicating shedding of the eGC. In this context, proinflammatory factors such as IL-1b, IL-6, TNF-a, MCP-1, and aldosterone are elevated in COVID-19 patients. Furthermore, the vascular leakage-inducing antagonist angiopoietin-2 and key markers for eGC shedding such as syndecan-1 and hyaluronic acid were significantly increased in COVID-19 patients [10, 11]. Heparanase activity is also enhanced, which is known to degrade the eGC and contribute to the inflammatory environment of the endothelium [12]. From these data, the hypothesis emerged that endothelial cells are activated and that the eGC is a highly vulnerable and crucial structure being specifically attacked during a SARS-CoV-2 infection and might determine the outcome of these patients.

From this point of view, the possibility of a therapeutic approach focusing on vascular eGC should be considered for COVID-19 patients. Heparanase inhibitors, matrix metalloprotease (MMP) inhibitors, or antioxidants could be discussed here [13]. Indeed, aldosterone levels are increased in COVID-19 patients, a condition leading to inflammatory processes, endothelial dysfunction, and vascular damage [11, 14]. Thus, the cytosolic aldosterone receptor, the mineralocorticoid receptor (MR), would represent another very promising therapeutic target.

However, these clinical observations and whether SARS-CoV-2 directly damages the endothelial surface are not fully understood yet. Here, by using the atomic force microscope [15] as a well-established nanoindentation tool [16, 17], we demonstrated that the eGC of primary endothelial cells after incubation with sera derived from COVID-19 patients with mild and severe symptoms is significantly damaged, which could be prevented by administering the MR antagonist spironolactone.

## Methods

### Human samples, demographics, and baseline characteristics

Blood samples of COVID-19 patients were collected from patients of the University Hospital Schleswig–Holstein Lübeck in cooperation with the Department of Infectious Diseases and Microbiology and the University Heart Center. COVID-19 patients were grouped into those with a milder course of disease and admitted to a general ward or with a severe course requiring intensive care and admitted to the intensive care unit. All sera were collected according to the Declaration of Helsinki and after approval of the local ethics committee (patients with a mild course of COVID-19: Az 13–003; patients with severe course of COVID-19: Az 19–019/A, University of Lübeck). All patients gave written informed consent. Sera were collected from female and male patients aged 30–78 years (mean ± SE: 58 ± 2.7 years, see Table 1) within 3 days of hospital admission. Serum was centrifuged at 2000 g and then stored at − 80 °C. Nonhospitalized, healthy, age- and gender-matched volunteers without comorbidities served as controls (hereafter termed “control”). These blood samples were collected at the University of Luebeck in cooperation with the Department of Cardiology and Angiology of the “Sana Kliniken Lübeck” hospital, Germany, following the Declaration of Helsinki and approved by the Local Ethics Committee (Case: 19–310).

**Table 1:** Demographics of control group, and C19 patient (mild and severe course)

Controls			Mild course			Severe course		
Control number	Gender	Age	Patient number	Gender	Age	Patient number	Gender	Age
*1*	m	81	*21*	m	45	*14*	m	56
*2*	m	75	*22*	m	47	*15*	m	62
*3*	m	50	*23*	m	52	*16*	w	56
*4*	m	82	*24*	m	70	*17*	m	64
*5*	m	46	*29*	m	71	*23*	w	58
*6*	m	47	*42*	w	30	*32*	w	47
*7*	w	53	*46*	w	51	*33*	m	66
*8*	w	62	*47*	m	64	*36*	w	78
*9*	m	49	*49*	w	43	*37*	m	70
*10*	w	61	*52*	m	70			
*11*	w	66	*58*	w	73			
*12*	w	66	*59*	m	44			

Controls were pooled for measurements.

### IL-6 quantification

IL-6 concentration (ng/L) was determined according to routine diagnostic testing at the University Hospital Schleswig–Holstein Lübeck. The accredited laboratory used an electrochemiluminescent immunoassay (ECLIA) to quantify IL-6 levels with a reference range of < 7.

### Cell culture

Primary human umbilical vein endothelial cells (HUVECs) were isolated (approved by the local ethical committee (Case: 18–325) and cultured as described previously [18, 19]. Cells were culture in HUVEC culture medium (Gibco Medium 199 + fetal calf serum 10% (Gibco, Carlsbad, CA) + penicillin/streptomycin 1% (Gibco, Carlsbad, CA; 100 U/ml; 100 mg/ml) + heparin 5000 U/ml (Biochrom, Schaffhausen, Switzerland) + large vessel endothelia supplement 1% (Gibco, Carlsbad, CA, USA). Cell culture flask were coated with 0.5% gelatin (1 h before seeding, Sigma-Aldrich, St. Louis, MO, USA) and cultivated at 37 °C, 21% O_2_ and 5% CO_2_. For AFM experiments, HUVECs were cultivated on fibronectin-coated glass coverslips to confluence for at least 4 days under standard cell culture conditions and stimulated with 10% COVID-19 sera or sera from a healthy donor group for 24 h before the experiment. The cells were co-treated with the MR antagonist spironolactone (100 nM) in parallel to COVID-19 sera treatment and compared to solvent control (0.1% ethanol).

### Nanoindentation measurements

The atomic force microscope (AFM, Nanowizard4 from JPK BioAFM Business, Berlin, Germany) was used to determine the nanomechanical properties of the eGC (here: eGC height) based on the nanoindentation technique as described previously [20]. Briefly, a laser beam was aligned on the backside of a gold-coated triangular cantilever (Novascan Technologies, Boone, NC, USA) with a mounted spherical tip (10 µm diameter) and a defined, nominal spring constant of 10 pN/nm. The cantilever indents the endothelial cell surface with a loading force of 0.5 nN. The reflection of a laser beam is used to quantify the cantilever deflection. The height of the eGC can be calculated by knowing the cantilever force, the piezo displacement, and the deflection sensitivity. The resulting force-distance curves were analyzed using the Protein Unfolding and Nano-Indentation Analysis Software PUNIAS 3D version 1.0 release 2.2 (http://punias.voila.net).

### Fluorescence staining

After 24 h stimulation with control/COVID-19 sera, eGC was stained by applying 2 µg/mL wheat germ agglutinin (WGA; conjugate Alexa-fluor-488; Thermo Fisher; Waltham, MA, USA) for 60 min in the dark (RT). Specimens were fixed with 4% fresh *paraformaldehyde* (PFA, 4 °C) for 30 min on ice. After washing the cells, the stained coverslips were mounted with Dako mounting medium (Dako, Carpinteria, CA, USA) with 1.5 µg/mL Hoechst (Sigma Aldrich, St. Louis, MO, USA) for nuclei staining. Fluorescence images were taken with a Keyence-BZ9000 fluorescence microscope (Keyence Corporation, Osaka, Japan). Fluorescence intensity was quantified using ImageJ software (Version 1.52a; National Institute of Health, USA).

### Statistical analysis

GraphPad PRISM (Version 7, GraphPad Software Inc., CA, USA) and IBM SPSS Statistics for Windows (IBM Corp. Released 2020, Version 27.0., NY, USA) were used to analyze the data and prepare the figures. Differences between experimental groups were analyzed using Student’s *t*-test for parametric values or (in case of three or more groups) assessed by using one-way ANOVA (analysis of variance) with Bonferroni’s correction for multiple comparison followed by post hoc Tukey’s multiple comparisons test for parametric values or the Kruskal–Wallis test by ranks followed Dunn’s multiple comparison for nonparametric values. For linear regression, Pearson (*r*) correlations were used. Differences were considered statistically significant when *p* values were < 0.05 (*p < 0.05; **p < 0.01; ***p < 0.001; ****p < 0.0001). Each experiment was repeated individually at least 3 times (*N* ≥ 3, independent HUVEC culture and stimulation) and each time with at least 1–3 technical replicates. The “*n*”-number summarized all replicates of this series (*n* ≥ 3–5). The *N*/*n* numbers are indicated in the figure legends.

## Results

### COVID-19 sera damage eGC structure

**Fig. 1:**
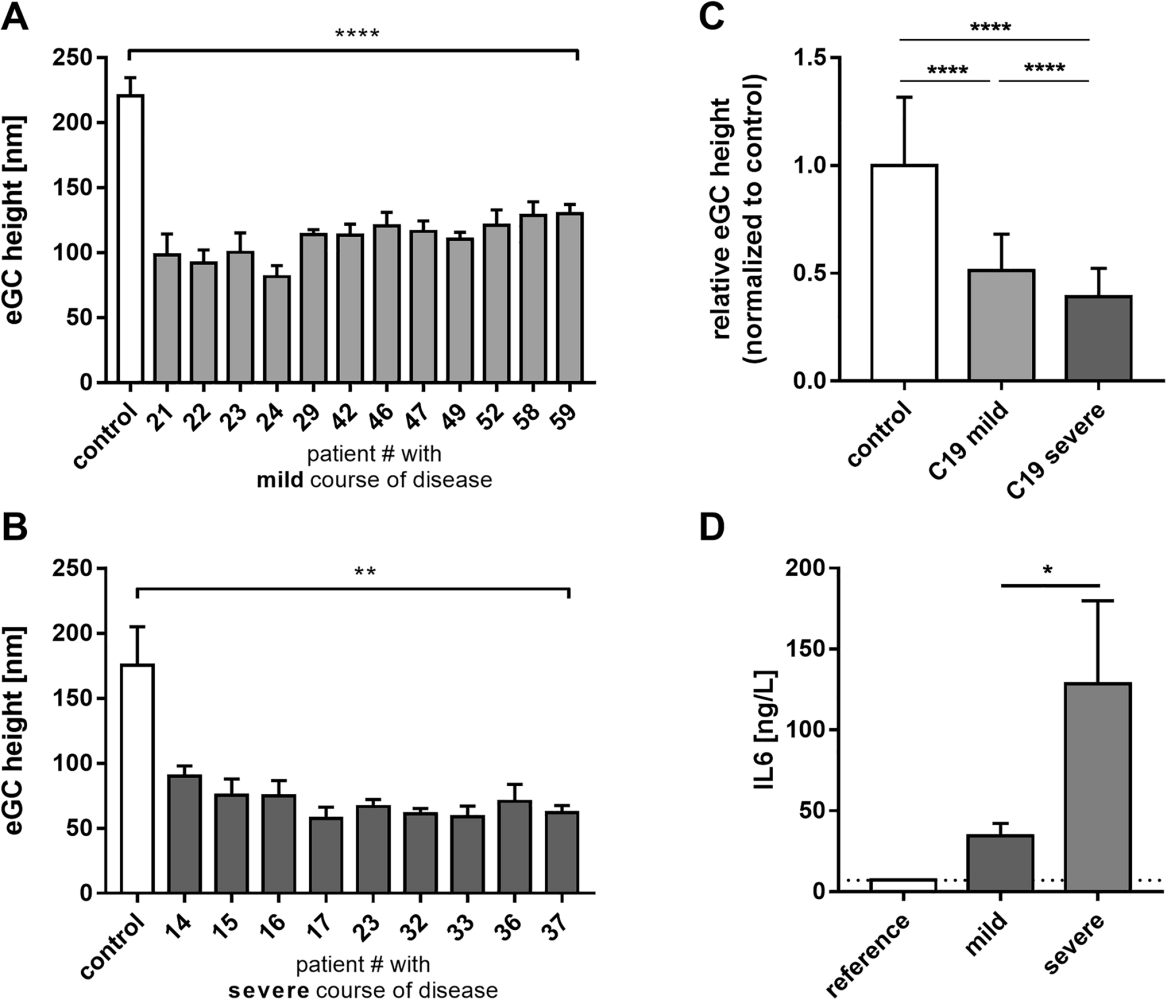
COVID-19 (C19) sera treatment damages the endothelial glycocalyx (eGC). **A** Endothelial cells were incubated with 10% sera from C19 patients with mild symptoms (patients 21–59) for 24 h. Analysis of the eGC by atomic force microscopy showed eGC damage with a reduced eGC height in a range of 81.6 nm to 130.1 nm compared to 220.7 nm of the control group (*N* = 3, *n* = 5–6; **** (all patients) *p* < 0.0001 vs. control). **B** Sera from a second patient cohort with severe SARS-CoV-2 infection and mandatory intensive care (patients 14–37) were incubated with endothelial cells for 24 h. Here, eGC height was further damaged, reducing eGC height in a range of 57.7 nm to 90.1 nm compared to 175.6 nm under control conditions (*N* = 3, *n* = 3–5; ** (all patients) *p* < 0.01 vs. control). **C** Average eGC height of control, C19 mild, and C19 severe are shown. C19 treatment leads to a reduction in eGC height by 48.9 and 60.8% compared to control in C19 mild and C19 severe, respectively (*N* = 3, *n* = 9–12, ****p < 0.0001). **D** IL-6 levels in mild and severe C19 patients were analyzed. In C19 patients, IL-6 increases compared to the healthy control reference level (7 ng/L). IL-6 was significantly higher in severe than in mild C19 samples

Confluent HUVEC monolayers were treated with 10% COVID-19 (C19) patient sera for 24 h and eGC height was quantified by using the nanoindentation AFM technique. Incubation with mild COVID-19 sera already decreased the eGC height in the range of − 41.1 to − 63.0% compared to control-treated HUVECs (see Fig. 1A). Treatment with severe COVID-19 sera augmented this effect, reducing the eGC height in the range of − 48.7 to − 67.2% compared to healthy controls (see Fig. 1B). After treatment with severe COVID-19 sera eGC damage was markedly stronger than after treatment with mild COVID-19 sera (see Fig. 1C; C19 mild overall mean 109.4 ± 3.4 nm vs. C19 severe overall mean 68.6 ± 3.2 nm, *p* < 0.001).

Levels of the proinflammatory cytokine IL-6 were analyzed in COVID-19 blood samples derived from patients with either mild or severe courses of disease (see Fig. 1D). IL-6 levels in samples derived from patients with severe COVID-19 course of disease were higher than from patients with mild symptoms (C19 mild: 34.3 ± 7.9 ng/L, C19 severe: 128.3 ± 51.5 ng/L, healthy reference: 7 ng/µL). Of note, patient IL-6 levels associated positively with eGC height (Pearson correlation: *r* = − 0.579, *p* = 0.024).

## Spironolactone treatment attenuates COVID-19-induced eGC damage

**Fig. 2:**
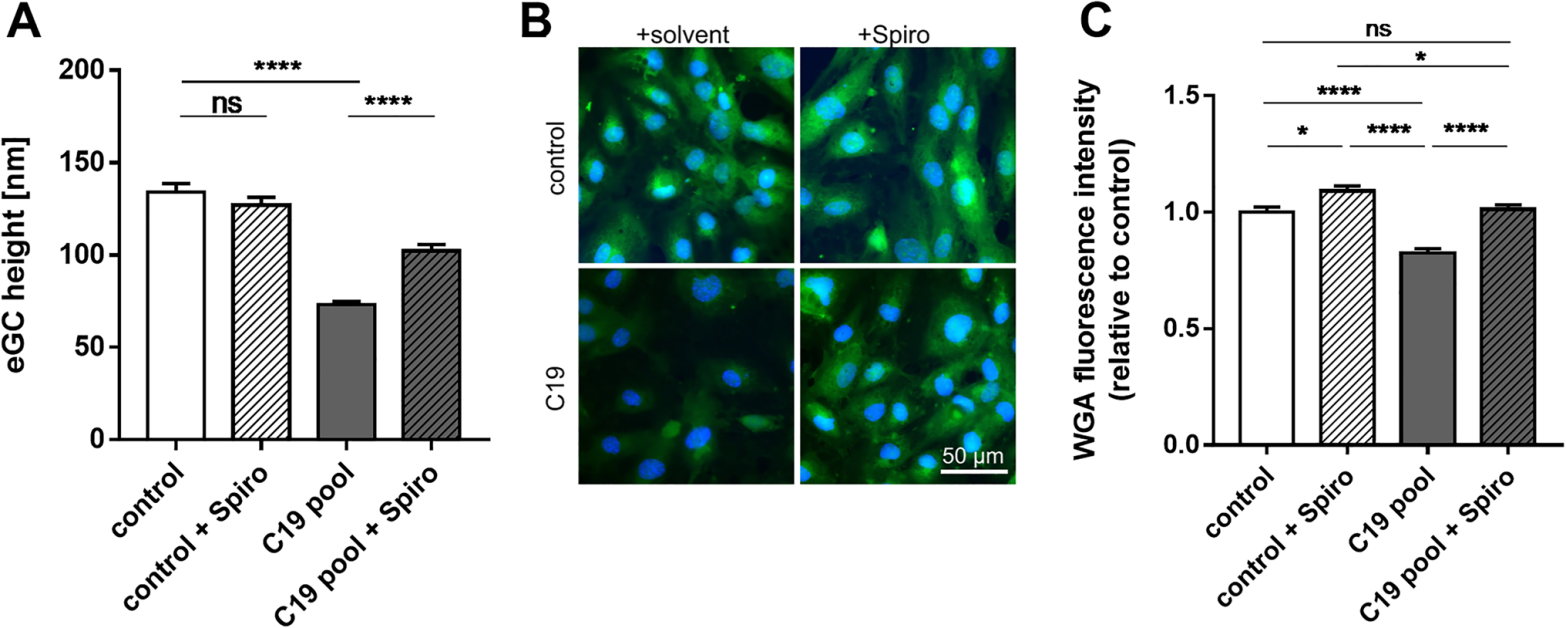
Treatment with spironolactone attenuates COVID-19 (C19)-induced endothelial glycocalyx (eGC) damage. **A** Sera from COVID-19 patients (mild course) were pooled and used for 24 h stimulation (10%) on endothelial cells. Treatment with C19 sera damaged the eGC and reduced height by 46% compared to treatment with healthy control sera. Coincubation with spironolactone strongly diminished the detrimental COVID-19 effect on eGC height within the C19-treated group (*N* = 3, *n* = 4–8; ****p < 0.0001). **B** Exemplary WGA stainings of HUVECs treated with 10% control or C19 sera with and without additional spironolactone incubation (100 nM). **C** WGA stainings were used to validate atomic force microscope measurements. Reduced eGC height in C19-treated cells was confirmed by decreased WGA fluorescence intensity compared to the control group. Spironolactone treatment strongly attenuated this effect (*N* = 3, *n* = 3–4, *p < 0.05, ****p < 0.0001)

Endothelial cells were treated with pooled sera from COVID-19 patients (mild courses) with or without coincubation with the MR antagonist spironolactone. Within the control group (sera from healthy donors), spironolactone treatment did not affect eGC height (control + solvent: 134.0 ± 4.7 nm vs. control + Spiro: 127.1 ± 4.1 nm). However, stimulation with pooled COVID-19 sera reduced the eGC height compared to control conditions by 46% (see Fig. 2A). Cotreatment with spironolactone strongly attenuates this effect, significantly improving eGC height within the mild COVID-19-treated group (C19 pool + solvent: 73.1 ± 1.9 nm vs C19 pool + Spiro: 102.2 ± 3.5 nm, *p* < 0.001).

Nanoindentation measurements were verified by eGC fluorescence staining with WGA (see Fig. 2B). Within the control group, spironolactone showed a slightly increased fluorescence signal compared to control conditions (Δ + 10.0 ± 2.1% compared to control, *p* < 0.05, see Fig. 2C). WGA staining confirmed COVID-19-induced eGC deterioration by reduced WGA fluorescence intensity in COVID-19 sera-treated cells (Δ − 18.6 ± 1.9% compared to control, *p* < 0.001). Coincubation of COVID-19 sera and spironolactone abolished changes in WGA fluorescence intensity compared to both the control and the pooled COVID-19 group.

## Discussion

In addition to pulmonary and cardiac effects, the vasculature is affected in COVID-19. This endothelial damage is multifactorial and complex, caused both directly by the SARS-CoV-2 virus and indirectly as a result of a systemic inflammatory cytokine storm [21, 22].

By using the AFM as nanosensor, our cell culture data suggests that in COVID-19, the endothelial surface is attacked and the eGC deteriorates in relation to disease severity (mild vs severe course) and in correlation to the IL-6 level. This deterioration could be prevented by administering low doses of spironolactone, a well-known and highly specific MR antagonist.

Early in the pandemic, the vascular endothelium was recognized as a crucial target of SARS-CoV-2 infections, including the notable progression of endothelial dysfunction, which persists at least over a period of 6 months [23]. Caused by circulating inflammatory mediators, the endothelial cells undergo a transition from a quiescent, functional to an activated, dysfunctional state, altogether making COVID-19 an endothelial disease [24]. For many years now, the eGC has been known to be a vulnerable structure on top of endothelial cells and a key regulator of endothelial cell homeostasis, tissue edema, and inflammatory processes [25]. Damage and deterioration of the eGC induce the development of endothelial dysfunction and cardiovascular pathologies [6].

Now, it has become clear that in SARS-CoV-2 infections especially the eGC is being attacked by proinflammatory cytokines, leading to shedding-related deterioration and loss of the vasoprotective function of the eGC [3, 13]. In agreement with these findings, the eGC damage in our patient cohort could be positively correlated to IL-6 levels.

From these data, it can be postulated that in COVID-19 patients increased levels of aldosterone [11] activate the NF-κB signaling pathway via the MR [26, 27], which activates the expression of pro-inflammatory cytokines such as TNFα and IL-6 [26]. This induces heparanase expression and subsequent degradation of the endothelial glycocalyx [28, 29] and could explain the mechanisms underlying the development of endothelial dysfunction under such conditions.

Recently, the eGC was found to be strongly involved in the attachment of viral spike-protein to the endothelial surface. Under healthy conditions, the eGC shields the spike-protein interaction with ACE2 receptors at the endothelial surface, whereas the ACE2 receptors are exposed after COVID-19-mediated eGC deterioration, which enables the binding of viral structures and subsequent infection of the cell [17]. In our study cohort, we could confirm that the eGC is severely damaged. Of note, the level of eGC damage depends on the severity of the disease course: sera derived from patients with mild symptoms reduced the eGC by approximately 49%, whereas sera from patients with severe symptoms reduced the height of the eGC by > 60%. This underscores clinical observations showing correlations between disease severity and syndecan-1 levels [30, 31].

To curb the effects of the COVID-19 pandemic, it is still imperative to find new therapeutic approaches. In this study, we demonstrated that by inhibiting the aldosterone receptor with low doses of spironolactone, COVID-19-induced damage of the eGC could be prevented.

Our in vitro data confirm these clinical observations. Aldosterone levels are increased in COVID-19 patients and the renin–angiotensin–aldosterone system (RAAS) can induce and modulate proinflammatory responses [11, 14]. Thus, it could play a key role in the pathophysiology of COVID-19.

Vicenzi et al. demonstrated that, in COVID-19 patients, treatment with the MR antagonist canrenone had an overall positive impact on all-cause mortality and clinical improvement, most probably via a direct anti-inflammatory effect [32]. In another study, it could be shown that SARS-CoV-2-induced endothelial injury was abrogated by the MR antagonist spironolactone, an FDA-approved drug [33].

The idea that MR antagonists have beneficial effects on the cardiovascular system emerged first nearly 20 years ago, when Pitt et al. demonstrated a reduced mortality and morbidity among patients with severe heart failure [34, 35]. Since then, hypotheses about the underlying mechanism of MR-dependent endothelial function and dysfunction, including effects on the eGC, have been developed [36].

In conclusion, we could demonstrate that proinflammatory mediators damage the eGC of COVID-19 patients in a severity-dependent manner: MR antagonist treatment could prevent this damage. We have identified (i) new mechanisms underlying COVID-19-mediated endothelial dysfunction and (ii) present a strategy for low-cost and effective medication to attenuate COVID-19 symptoms and to prevent cardiovascular damage. Importantly, this could also be beneficial for developing and emerging countries and independent of variations and mutations of the virus.

## Data Availability

The datasets generated during and/or analyzed during the current study are available from the corresponding author on reasonable request
